# Community engagement and the centrality of ‘working relationships’ in health research

**DOI:** 10.1136/bmjgh-2024-015350

**Published:** 2024-04-24

**Authors:** Robin Vincent, Dorcas Kamuya, Bipin Adhikari, Deborah Nyirenda, James V Lavery, Sassy Molyneux

**Affiliations:** 1 Robin Vincent Learning and Evaluation LTD, Leeds, UK; 2 Health Systems and Research Ethics, Kemri-Wellcome Trust Research Programme, Nairobi, Kenya; 3 Nuffield Department of Population Health, University of Oxford, Oxford, UK; 4 Nuffield Department of Medicine, University of Oxford, Oxford, UK; 5 Mahidol University, Salaya, Thailand; 6 Community Engagement & Bioethics, Malawi-Liverpool-Wellcome Trust Clinical Research Programme, Blantyre, Malawi; 7 Emory University, Atlanta, Georgia, USA; 8 Health Systems and Research Ethics, Oxford University, Oxford, UK

**Keywords:** Global Health

## Introduction

Community engagement (CE) is widely accepted as a critical aspect of health research because of its potential to make research more ethical, relevant and well implemented. Although CE activities linked to international health research involving Low and Middle Income Countries (LMICs; see author note) have proliferated and are increasingly described in published literature, there is a lack of conceptual clarity around how engagement is understood to ‘work’.^1^ Ultimately, the evidence base for CE remains underdeveloped,^2^ despite increasing scholarship. We conducted a realist review–a theory-driven approach to evidence synthesis—to better understand the causal dynamics of CE practices associated with health research in LMICs.^3^ We selected large malaria trials as the entry point for the review because there is a well-established tradition of CE in malaria research, and because this area provides a good representation of current CE practice in international biomedical research involving LMICs. In this commentary, we summarise and discuss the key findings and implications of the review.

## Working relationships as the core of CE

**Figure 1:**
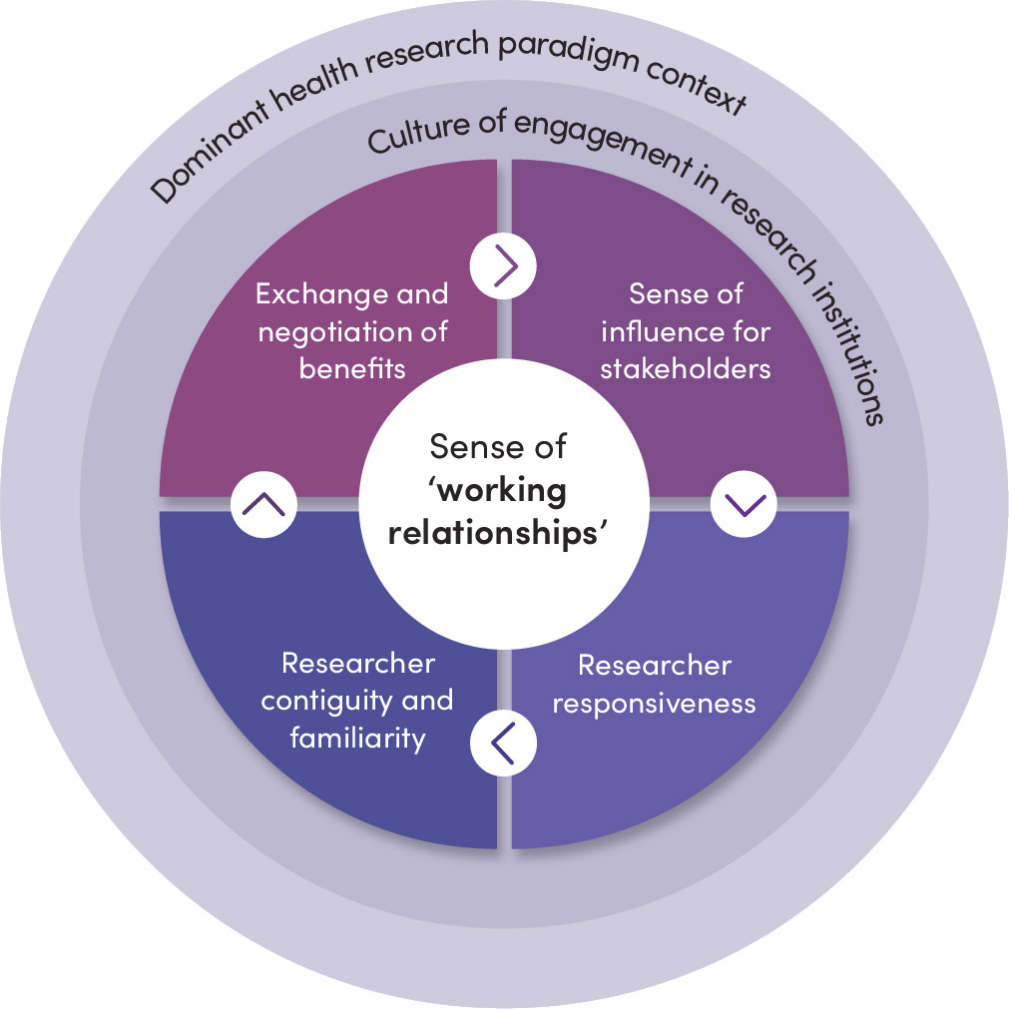
Core Community engagement dynamic of working relationships and influences of context.

Our review highlighted that at the core of CE in health research is the establishment of ‘working relationships’ between researchers and other local stakeholders, influenced by the culture of engagement in research institutions, in turn, shaped by the wider health research paradigm context (figure 1, adapted from Vincent *et al* 3).

The centrality of working relationships underscores the importance of relational dynamics in CE. Working relationships are influenced by differences between and among researchers and other local stakeholders in wealth, power and culture, and depend on four mutually reinforcing relational dynamics:

- Exchange and negotiation of mutual benefits from research. Such benefits for researchers include samples, data and potential for career progression. For participating families and communities, benefits include access to quality healthcare, financial support and new network members.
- Researcher responsiveness to stakeholder interests, including the degree that they feel listened to, and their concerns addressed.
- ‘Contiguity’ or sense of everyday presence and accessibility of research staff, especially through research ‘fieldworkers’ and other ‘front-line’ staff who are often from and regularly interact with community members.
- A sense of influence over some aspects of the research process by stakeholders.

A combination of concrete benefits and the experience of reciprocal relationships are central to how CE works, rather than any particular engagement activity (such as a community advisory board meeting) or method (such as a community meeting). Developing working relationships can contribute to acceptance and participation in research among some community members, even where research staff and community members have different interests in participating. Importantly, however, relationships can be precarious and are constantly shifting.

## Working relationships are shaped by the ‘terms of engagement’

Working relationships are shaped by ‘terms of engagement’. The notion of ‘terms of engagement’ draws on insights from scholarship on ‘spaces of participation’^4^ and the sociology of power^5^ to include the implicit expectations and conventions developed through previous experience that set the scene for, and frame, current interactions. Our analysis found that the ‘terms of engagement’ that shape working relationships—including the background conditions under which people participate in research—are influenced by key characteristics of the dominant health research paradigm, set largely by high-income country (HIC) research institutions and funders to align with their own strategic goals, operating procedures and workflows.

Health research in LMICs, such as the malaria trials in our review, tends to be funded and governed primarily by international partnerships, increasingly deployed through an international infrastructure of trial-specific organisations and agencies. This limits national governments’ interest and ability to set and follow local research agendas.^6^ It also means, in practice, that the main power holders in research remain the institutions and researchers based in HICs, with LMIC-based researchers having less decision-making power, and less access to and control over research infrastructure and facilities.^7^ These power dynamics are reproduced through interactions at regional, local and institutional levels—favouring those with the strongest external connections and networks with the greatest decision-making power and authority.

In large malaria trials, community members and other local research stakeholders rarely have significant influence on research agendas or protocols despite aspirational claims about community empowerment and country ownership. Indeed, much collaborative research in ‘global health’ takes place against a historical background of colonialism and in the context of donor-funded vertically delivered health programmes and research investments with a poor record of building public health and research infrastructure.^8^ The coloniality of knowledge production across global health research and practice is increasingly recognised and challenged.

## ‘Working relationships’ as mechanisms for change?

Our analysis suggests that the current practices and dynamics of CE, paradoxically, tend to perpetuate the problematic features of the dominant health research paradigm, rather than counter them. These seemingly contradictory findings have also been observed in global health partnerships, many of which involve collaborative research between HIC and LMIC institutions. A review by Plamondon *et al*,^9^ for example, found that global health partnerships ‘can serve to entrench both inequitable relationships and unfair distributions of power, resources, and wealth within and between countries (and partners) if inequitable power relationships are left unmitigated’.

Against the grain of our analysis overall, a small amount of literature highlights how an ethos of respect for stakeholders and commitment to shared decision-making in CE can set in motion interactions and relationships that become a new ‘equity context’^10^ that encourages further collaboration and engagement. This more collaborative dynamic was identified in some cases where malaria was tackled as part of a comprehensive primary healthcare intervention—where there was more emphasis on local stakeholder control over priority setting and involvement in problem-solving in relation to local health challenges.^11^ It was also identified in cases where malaria trials were being conducted as part of longer and deeper research partnerships with long-term commitment and investment in interdisciplinary capacity building of national researchers and institutions, and where research institutions and senior researchers valued engagement and committed the necessary resources to support engagement activities. Illustrations of resources committed include dedicating specific research team members as points of contact for stakeholders; ensuring career pathways, training and support for front-line research staff; drawing on technical inputs from social scientists and other experts and investment in processes of reflection and evaluation that inform institutional processes.

Although the importance of relationships for equitable partnerships was noted, our review did not provide a strong enough signal to identify relationships as critical mechanisms and vehicles for identifying and addressing equity-related challenges that arise in partnerships. Nevertheless, our analysis suggests that the ‘working relationships’ between researchers and other stakeholders established through CE may provide insights about stakeholder interests and partnerships that can be drawn upon to support fairer partnerships.^2^ Even then, CE activities and insights can only be part of a wider and deeper set of initiatives to transform power relations in global health.

## Widening the focus of CE

Our analysis highlights the crucial role of access to healthcare and the under-resourcing of health systems in CE relational dynamics, and the importance of widening the focus of CE beyond individuals and immediate research relationships to better understanding and challenging broader health systems and social, political and economic inequities.^12 13^ The meso level context such as the facilities and institutions involved in health research and delivery, as well as the social, political and economic influences on health and research systems, are therefore essential research areas. More explicit attention also needs to be paid to how the resources accompanying large health research trials affect health and research systems in the short, medium and longer term.

Our review provides examples of studies and sets of ‘working relationships’ created through CE that contribute (although in limited ways) to strengthening local health infrastructure, health surveillance and improvements in staffing levels, supplies and diagnostics. A greater appreciation of the insights that can arise from such efforts could open up new opportunities for HIC and LMIC institutions to more systematically design and implement collaborative health research strategies that intentionally strengthen—and avoid undermining—local research ecosystems and health systems.

## Conclusion

Viewing CE as a ‘human infrastructure’^14^ of ‘working relationships’—structured by unequal ‘terms of engagement’ between the different stakeholders involved—can generate valuable insights for improving research partnership practices. Greater incorporation of theoretical understanding of the relational dynamics of engagement, including complex power relations within and between stakeholder groups, can underpin better engagement strategies and facilitate the kind of responsive interactions that are key to developing productive working relationships. And greater attention to the nature of the insights generated through working relationships could reinforce the unique knowledge-generation capabilities of CE, in addition to the more obvious advantages in building mutual respect and understanding that are essential for any well-functioning partnerships. The findings from our review offer a new way of conceptualising the role of CE in research partnerships. The challenge now is to encourage the kind of self-reflection at both individual and institutional levels, in both HIC and LMIC institutions, required to reap the potential value of insights generated through the ‘working relationships’ of CE.

## Data Availability

No data are available.
